# Ethnopharmacological Value and Biological Activities via Antioxidant and Anti-Protein Denaturation Activity of *Morinda lucida* Benth and *Momordica charantia* L. Leaves Extracts from Benin

**DOI:** 10.3390/plants12061228

**Published:** 2023-03-08

**Authors:** Durand Dah-Nouvlessounon, Michaelle Chokki, Agossou Damien Pacôme Noumavo, Geta Cârâc, Bianca Furdui, Haziz Sina, Cheikna Zongo, Aly Savadogo, Lamine Baba-Moussa, Rodica-Mihaela Dinica, Farid Baba-Moussa

**Affiliations:** 1 Laboratory of Biology and Molecular Typing in Microbiology, Department of Biochemistry and Cell Biology, Faculty of Sciences and Technic, University of Abomey-Calavi, Cotonou 05BP1604, Benin; dahdurand@gmail.com (D.D.-N.); pacome.noumavo@gmail.com (A.D.P.N.);; 2 Department of Chemistry, Physics and Environment, “Dunarea de Jos” University of Galati, Domneasca Street 47, 800008 Galati, Romania; michaellechokki@gmail.com (M.C.); getacarac@ugal.ro (G.C.);; 3 Laboratoire de Microbiologie et de Technologie Alimentaire, FAST, Département de Biologie Végétale, Université d’Abomey-Calavi, ISBA-Champ de Foire, Cotonou 01BP: 526, Benin; 4 Centre de Recherche en Sciences Biologiques, Alimentaires et Nutritionnelles (CRSBAN), UFR-SVT, Université de Ougadougou, Ougadougou 03BP7131, Burkina Faso

**Keywords:** anti-inflammatory activity, volatile compounds, plants extract, cyclic voltammetry, GC-MS analysis, natural products

## Abstract

*Momordica charantia* Linn. (Cucurbitaceae), the wild variety of bitter melon, and *Morinda lucida* Benth (Rubiaceae) were commonly used as a popular folk medicine in Benin. This study aimed to appreciate the ethnopharmacological knowledge and evaluate the antioxidant and anti-inflammatory effects of *M. charantia* and *M. lucida* leaves extracts. Semi-structured surveys supported by individual interviews were conducted with herbalists and traditional healers in southern Benin. The antioxidant activities were evaluated by a micro-dilution technique using ABTS and FRAP methods. These activities were supported by cyclic voltammetry analysis. The anti-inflammatory activity was evaluated by the albumin denaturation method. The volatile compounds were analysed by GC-MS analysis. All the respondents involved in this study have good knowledge of the two plants. We identify 21 diseases grouped into five categories of condition. The two plants’ extracts possess variable antioxidant capacity. Indeed, all the active extracts of *M. charantia* presented an IC_50_ < 0.078 mg/mL, while the extracts of *M. lucida* had an IC_50_ up to 0.21 ± 0.02 mg/mL. For anti-inflammatory activity, a dose-response activity (*p <* 0.001) was observed in the protein denaturation inhibition rate of the extracts. It should be noted that the highest inhibition rate (98.34 ± 0.12) of the albumin denaturation was observed with *M. lucida* dichloromethane extract. A total of 59 volatile compounds were identified by GC-MS analysis in the extracts of the two plants. The *M. charantia* ethyl acetate extract shows the presence of 30 different compounds with a relative abundance of 98.83%, while that of *M. lucida* shows 24 compounds with a relative abundance of 98.30%. These plants are potential candidates to discover new compounds with therapeutic properties that could be used to solve public health problems.

## 1. Introduction

Africa is composed of several hundred different ethnic and linguistic groups who coexist in a highly diversified tropical environment. These different ethnic groups have very important knowledge used for their survival and to meet their health needs. Indigenous knowledge related to medicinal plants is a foundation of primary health care for treatment of several human and animal diseases among local communities, especially in developing countries [1,2]. However, nowadays, natural resources are considered very important, but they are insufficiently known. The vegetation is distributed in innumerable ecosystems; that of the humid forest and coastal regions has an impressive atmosphere and exuberance due to an extremely rich flora. Nearly 50,000 species of plants have been counted in Africa out of the 250,000 species existing throughout the world. Evidence indicates that up to 80% of the population in developing countries use herbal medicines as the primary form of healthcare [3]. This could also justify the fact that in Africa, people have always traditionally had very rich knowledge thanks to the cultural and ecological diversity of the environment in which they live [4]. This knowledge is usually the sum of daily experiences and the knowledge of ethnic groups that forms the basis for decision-making in the face of health and livelihood issues. Thus, in several countries of the continent, plants constitute the main medicinal means for practical care in public health. In Benin in particular, the flora abounds in a diversity of plants used for food, crafts, cultural and medicinal purposes [5]. Among the species listed are *Momordica charantia* from the Cucurbitaceae family, and *Morinda lucida* from the Rubiaceae family.

In Benin, both plants are used for the treatment of diabetes. Our previous work has proven the effectiveness of these two plants in the treatment of diabetes [6]. Other authors have also proven the use and therapeutic virtues of these two plants [7,8,9,10,11,12,13].

Inflammation is considered an important etiological factor in the development of both types 1 and 2 diabetes mellitus [14,15]. An excessive increase in reactive oxygen species (ROS) or reactive nitrogen species (RNS) accompanied by a decrease in antioxidants induces oxidative stress, which can lead to endothelial dysfunction, insulin resistance and alterations in the number of reactive oxygen species and function of pancreatic beta cells [16]. Therefore, phytochemicals with hypoglycemic, anti-inflammatory and antioxidant activities are suited for the treatment of diabetes [17].

This study aimed to improve the level of scientific knowledge on these species through an ethnopharmacological survey, and to elucidate the biological activity through the determination of the chemical composition of the two plants extracts with gas chromatography-mass spectrometry (GC-MS). Diverse biological activities, including anti-inflammatory and antioxidant activities, were evaluated. The effect of the reversibility of the antioxidant activity was evaluated by electrochemistry using cyclic voltammetry.

## 2. Results

### 2.1. Ethnopharmacological Survey of Medicinal Use of M. charantia and M. lucida

#### 2.1.1. Socio-Cultural Characteristics of Respondents

The ethnopharmacological surveys were carried out with 137 herbalists and traditional healers, including 20.44% men and 79.56% women. The age of the respondents varied between 18 and 65 years old. Respondents whose age was between 25 and 50 represent the vast majority, i.e., 52% of respondents. The respondents were divided into five ethnic groups of which the “ouémè” ethnic group is the majority (31.38%) and the “sèto” ethnic group is the minority ethnic group (8.75%).

#### 2.1.2. Knowledge of Plant Material

All the respondents involved in this study have good knowledge of the two plants (*Momordica charantia* and *Morinda lucida*). The recognition criteria are essentially based on botanical characteristics, such as the appearance and shape of the plants, leaves and fruits. Indeed, the main recognition criterion is that *M. charantia* is a liana, while *M. lucida* is a plant that can reach 12m in height with large leaves. Both species are common in the study area, but there is a period of abundance (the rainy season) for *M. charantia*. On the other hand, *M. lucida* is available during both seasons of the year. Both species are found in their natural habitat, as well as on farms and in gardens.

#### 2.1.3. Ethnopharmacological Data Related to the Use of the Two Species

The two study plants are used in the treatment of human and animal diseases. Indeed, from the surveys, we identify 21 diseases grouped into five categories of conditions (digestive system diseases, microbial and parasitic diseases, gynaecological diseases, blood-related diseases and others). Some diseases are common to both plants species, while others are specific to each species. Among the common diseases treated, we have: malaria, microbial and parasitic infections, diabetes, inflammation, hypertension and strengthening of immunity. Table 1 presents the frequency of the diseases treated with *M. charantia* and *M. lucida*. For *M. charantia*, the most treated disease is measles (100%), followed by dermatological problems with wounds included (98.54%) and diabetes (71.53%). For *M. lucida*, malaria is the most cited disease (93.43%), followed by abdominal pain (74.45%) and diabetes (56.93%).

**Figure 1:**
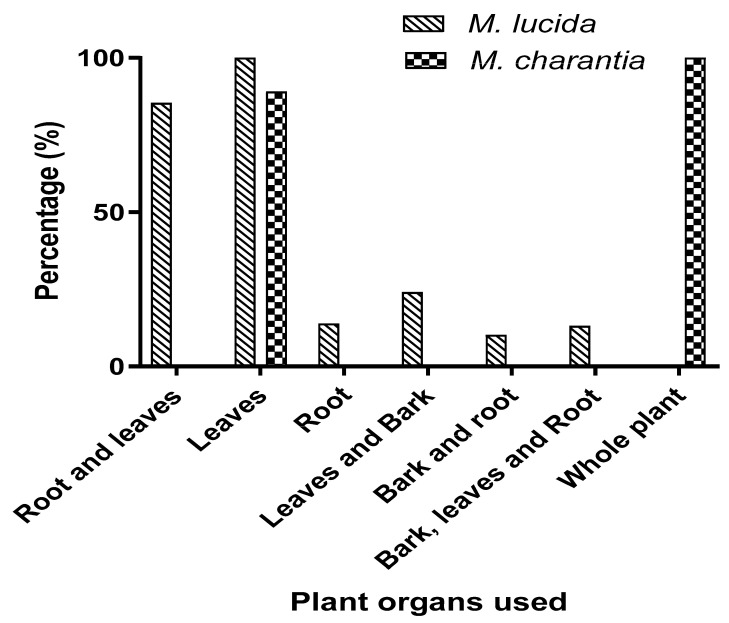
Proportion of parts of plant used alone or in combination.

In addition, different organs of the two plants are used either alone or in association with other plants. Indeed, the use proportions of each organ of the same plant in the formulation vary according to the plant and the diseases treated. For *M. lucida*, the leaves are the most used organs, followed by leaf + root mixtures. For *M. charantia*, only the leaves or the whole plant are used (Figure 1).

As we mentioned above, the two species of plant are used in the composition of recipes for the treatment of certain diseases. Information concerning the type of recipe, the solvent used, the parts of the plant used, the method of preparation and the dosage are grouped in Table 2 and Table 3.

### 2.2. Antioxidant Activity of M. charantia and M. lucida Extracts

#### 2.2.1. ABTS Method

Table 4 presents the results of the antioxidant activity with the ABTS method. *M. charantia* and *M. lucida* extracts possess variable antioxidant capacity for ABTS inhibition. The highest percentage inhibition for the active extracts of *M. charantia* (50.52 ± 1.20%) was obtained with the acetone extract, while the lowest (3.86 ± 0.11%) was obtained with the methanolic extract. For *M. lucida*, the greatest percentage inhibition (48.27 ± 1.54%) was obtained with the ethanolic extract, while the lowest percentage inhibition (4.63 ± 1.41%) was obtained with the acetone extract.

Methanol/HCl-PE: Methanol/HCl extract from the extraction of the residue obtained after extraction with petroleum ether. Methanol-EA: Methanol extract from the extraction of the residue obtained after extraction with Ethyl Acetate.

#### 2.2.2. Ferric Reducing Antioxidant Power (FRAP)

Table 5 presents the reducing power of Fe^3+^ ions by *M. charantia* and *M. lucida* extracts. Regarding ferric reducing antioxidant power, the obtained results indicate that higher sample concentrations (5 mg/mL) cause a greater value of inhibition percentage of the active extracts. For *M. charantia*, the highest inhibition value (60.75 ± 1.07%) was obtained with an ethanol–water extract, while with *M. lucida*, the methanol extract shows the highest value (60.24 ± 0.56%) of inhibition (Table 5). In comparison of the activity of the two plants, the same trend was observed with the IC_50_. Indeed, all the active extracts of *M. charantia* presented an IC_50_ < 0.078 mg/mL, while the extracts of *M. lucida* had an IC_50_ up to 0.21 ± 0.02 mg/mL. However, the comparison of the effect of the extracts shows that only the ethanol–water extract of *M. charantia* (60.75 ± 1.07%) is higher (*p =* 0.014) than that of *M. lucida* (58.23 ± 1.26%).

### 2.3. Cyclic Voltammetry Analysis of M. charantia and M. lucida Extracts

**Figure 2:**
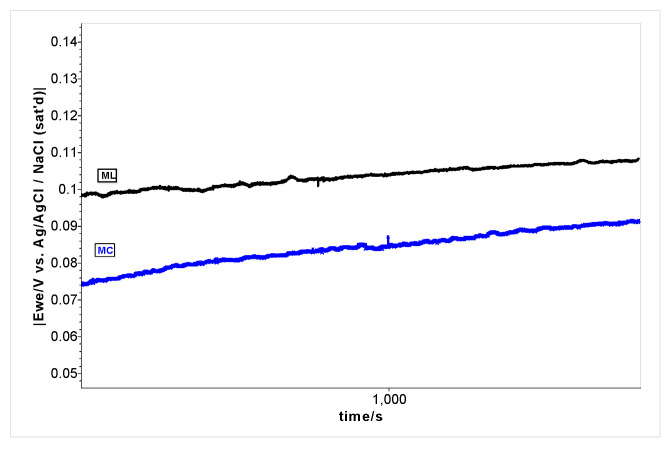
Open circuit potential (OCP) registered for *M. charantia* (MC) and *M. lucida* (ML) samples in methanol for 30 min.

The antioxidant activity potential of the plants’ leaf extracts in methanol using cyclic voltammetry technique has been investigated. Initial, the open circuit potential (OCP), which measures the equilibrium potential at the electrode surface, was registered (Figure 2) and can be used to quantify a samples’ redox potential, which is a function of its composition. The lower positive potentials for the *M. charantia* samples were from 0.074 ± 0.002 V, which increase up to 0.092 ± 0.003 V after 30 min. Compared with *M. charantia*, for the *M. lucida* samples, the potential started from 0.093 ± 0.003 V and increased up to 0.113 ± 0.008 V. These data indicate the overall redox potential of both plant extracts in methanol without any significant difference.

**Figure 3:**
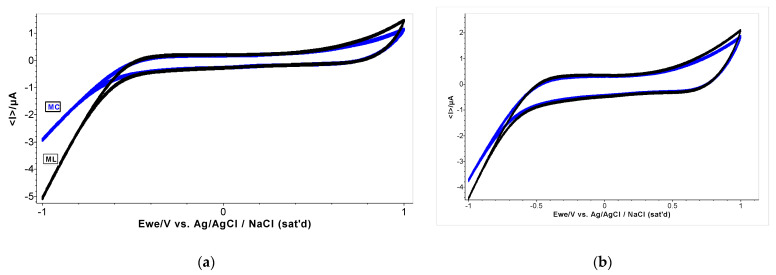
Cyclic voltammogram at scan rate 0.100 V s^−1^ (**a**) and at scan rate 0.200 V s^−1^ (**b**) for *M. charantia* (MC) and *M. lucida* (ML) in methanol without electrolyte support.

Both systems were further investigated using the cyclic voltammetry (CV) technique at variable scan rates, between 0.01–0.2 V·s^−1^. The cyclic voltammograms registered for *M. charantia* and *M. lucida* in methanol are presented in Figure 3 at the scan rate of 0.01 V·s^−1^ and the scan rate of 0.2 V·s^−1^. The observed behaviour of the samples shows the electro-lower-inactivity of the methanol extracts in the selected potential range from −0.50 V to +0.35 V. The antioxidant capacity of the extract is highlighted by its redox profile from CV. There was an increase of the oxidation peak current with increasing scan rate. The increase in the anodic current is attributed to the presence of some active components (antioxidants) in the extracts. The additions of 1 mL plant extract were made volumetrically as the concentration of the compounds of extracts was not possible to be calculated. In the *M. lucida* sample, the anodic current is 1.63 μA at the scan rate of 0.200 V·s^−1^, and depending on the scan rate applied in general, it increases. In the *M. charantia* sample, the anodic current is 1.86 μA at the scan rate of 0.200 V·s^−1^. A linear relationship was obtained between the cathodic peak current and the square root of the scan rate, suggesting a diffusion-controlled electrode process for oxygen reduction in methanol.

**Figure 4:**
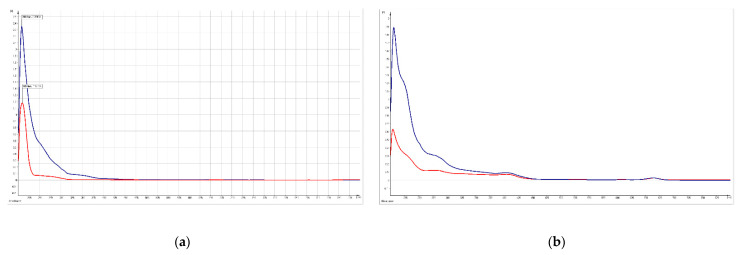
UV-Vis spectra registered for the *M. charantia* (**a**) and *M. lucida* (**b**) samples before and after the cyclic voltammetry experiment.

Both anodic areas of the cyclic voltammograms show the total content of the antioxidant compounds, allowing discrimination between the different extracts. We can conclude that the extracts undergo an irreversible oxidation in the same conditions, for the anodic peak, with the current increasing with different values (13.3 μA for *M. charantia* and 3.08 μA for *M. lucida*). However, of more interest is the cathodic current registered for the samples, when the values are bigger than the anodic current up to -10 μA, and lower at a smaller scan rate. Reduction peaks were observed at all scan rates and shifted to a more negative value as the scan rate decreased in both plants’ systems. These dates confirm that all reduction compounds are active species working for redox exchange and the resulting oxidants could be active again for new electronic activity. The CV technique can also be helpful to determine the mechanism of free radical scavenging activity because of their potential use in traditional medicine. The results are consistent with the spectroscopic results, which emphasize that plant extracts have no different antioxidant compounds (Figure 4).

### 2.4. Anti-Inflammatory Activity of M. charantia and M. lucida Extracts

The results of protein denaturation inhibition are shown in Table 6. The protein denaturation inhibition rate of the extracts at different concentrations increases with increasing extract concentration. A dose-response activity is therefore observed. For *M. charantia*, the inhibition rates vary from 93.09 ± 1.17% (aqueous extract) to 99.53 ± 0.08% (methanolic extract). Although the methanolic extract presented the highest percentage of inhibition, it was the dichloromethane extract that presented the lowest IC_50_ (0.10 ± 0.02 mg/mL), thus showing the strongest ability to inhibit albumin denaturation. Contrary to the observation made with *M. charantia*, it is the aqueous extract of *M. lucida* that presented the lowest IC_50_ (0.11 ± 0.01 mg/mL), thus showing the strongest activity of the extracts of *M. lucida*. However, it should be noted that the highest inhibition rate (98.34 ± 0.12) of the albumin denaturation was observed with the dichloromethane extract. The interaction between the extracts and the plants is variable (*p* < 0.001). The compared effect of *M. charantia* extracts shows a difference between the inhibition rate of the aqueous extract with the ethanol, methanol, dichloromethane (*p* < 0.001), ethyl acetate and acetone (*p* = 0.033) extracts. A variation in the inhibition rate was also observed with the *M. lucida* extracts, with the lowest variation between the ethyl acetate and chloroform extract (*p* < 0.05).

### 2.5. Gas Chromatography Coupled with Mass Spectrometry (GC-MS)

GC-MS was performed on the ethyl acetate and acetone extracts of each plant species (*M. charantia M. lucida*). The results show that the quality and the quantity of the volatile compounds vary according to the extracts and the plants (Table 7). A total of 59 volatile compounds were identified and quantified in the extracts of the two plants.

For *M. charantia*, the GC-MS analysis of the ethyl acetate extract shows the presence of 30 different compounds with a relative abundance of 98.83%. The main compounds in this extract are: 2,6-Bis (1,1-dimethylethyl)-4-(1-oxopropyl)phenol (27.03%), followed by Ethyl iso-allocholate (14.33%), 3,9,14,15-Diepoxypregn-16-en-20-one, 3,11,18-triacetoxy (11.26%) and Benzothiophene-2-carboxylic acid, 4,5,6,7- tetrahydro-7-hydroximino-3-[2-(4-morpholyl)-1-oxoethylamino]-, ethyl ester (10.54%), which have at least a relative abundance of 10%. The GC-MS analysis of the acetone extract shows the presence of 23 different compounds with a relative abundance of 99.03%.

For *M. lucida*, the GC-MS analysis of the ethyl acetate extract shows the presence of 24 different compounds with a relative abundance of 98.30%. On the other hand, that of the acetone extract reveals the presence of 25 volatile compounds with a total relative abundance of 96.49%. The most abundant compounds in this extract are: N,N’-Bis(Carbobenzyloxy)-lysine methyl (ester) with an abundance of 22.84%, followed by 17-Hydroxy-3,20-dioxopregna-1,4, 9(11)-trien-21-yl acetate (20.02%) and 6β-Hydroxyfluoxymesterone (14.31%).

## 3. Discussion

The ethnopharmacological studies carried out made it possible to enroll a total of 137 herbalists and traditional healers, including 20.44% men and 79.56% women. All of the herbalists are women, and 100% of traditional healers are men. This could be explained by the fact that in Benin, selling in public markets is much more an activity reserved for women. The main respondents are therefore women from rural areas and are mostly illiterate. Indeed, according to several authors, rural populations, mostly illiterate, possess medicinal knowledge of plants [18,19]. Rehman et al. [18] reported that in tribal communities in Pakistan, traditional healers possess a lot of information about medicinal plants. In these regions, medicinal plants are important for the indigenous people, providing access to basic healthcare [20]. In our study, this illiterate nature of the respondents is also observed by other authors [21] who affirm that the use of medicinal plants remains the main means of treatment for people of a certain social class. The age of the respondents varied between 18 and 65 years old. However, respondents whose age was between 25 and 50 represent the vast majority, i.e., 52% of respondents. It is especially accepted in Africa that they are the wise men, the people of a certain age, who hold the traditional knowledge of treating illnesses. In addition, the medicinal virtues of plants are ancestral knowledge that is transmitted from generation to generation [22]. This is justified by the proportion of 64.23% of respondents who have experience of between 16 and 30 years in the field of medicinal plant exploitation. This seniority in the field of the exploitation of medicinal plants confirms the richness of the information collected with consensus factors very close to the value 1. A consensus factor close to this value shows the degree of homogeneity and accuracy of the information [23]. Both plant species are used in the treatment of several diseases, such as diabetes, cancer, infectious diseases (microbial and viral infection) and inflammatory diseases, to treat chronic wounds and to strengthen immunity. Some authors in other countries in Asia [24], America [25] and the West African sub-region [26,27,28] had already reported the efficacy of these two species in the treatment of these diseases. Lakouéténé et al. [29] reported that a species can be used for one or more pathologies. It is evident through the results of this ethnopharmacological study that the two plants are used by the study population to effectively treat several pathologies. In this context, research must accompany this attitude of the population for better management of diseases. For both species, the leaves are the most used organs for the treatment of these diseases. These observations have also been made by other authors, such as [30,31]. Indeed, the intense picking of the leaves does not present any danger to the plant. According to Ouattara [32], removing 50% of a tree’s leaves does not significantly affect its survival. In addition, various methods of preparation (decoction, maceration, and trituration) have been observed in the use of these plants. Decoction is used more than the other methods of preparation. Bla et al. [30] also noticed that this mode of preparation is used more than the others. In addition, the organs of these plants are used in the formulation of several recipes. *M. charantia* and *M. lucida* are used for specific mono recipes or in association with other plants. Mono-specific recipes were in the majority compared to plant associations. This trend is confirmed in other studies [32], who reported 24 medicinal recipes, 21 of which are mono-specific, i.e., 87.5%. The phytochemistry of plants using GC-MS revealed the richness of the extracts of the two plants in volatile compounds through the identification of more than 58 volatile compounds with variable relative abundances. 2,6-Bis (1,1- dimethylethyl)-4-(1-oxopropyl)phenol (27.03%) was the more abundant compound found in the both extracts. This compound was also identified in jujube fruits by Wang et al. [33] with good antioxidant activity. Zeb [34] also reported its antioxidant activity in edible oils. Apart from this compound, ethyl iso-allocholate was also found in all the extracts of both plants with a relative abundance of 14.33%. Other authors, including Haider et al. [35] and Jasso de Rodríguez et al. [36], isolated this compound in other plant species, *Adiantum capillus*-Veneris and *Psacalium paucicapitatum*, respectively. This compound (Ethyl iso-allocholate) of steroidal nature has been reported as a potential therapeutic agent, as it has diuretic, anti-inflammatory, anticancer and antimicrobial activities [35,36,37]. Chokki et al. [6] also reported that the antimicrobial activity observed with these plants would be attributed to their chemical composition.

Other studies [38,39] have shown that these plants contain various active metabolites known to have many biological activities. The extracts of *M. charantia*, as well as those of *M. lucida* prevented the denaturation of the protein used in this study at percentage inhibitions ranging up to 99.53±0.08% for the methanolic extract of *M. charantia*. Among these metabolites, flavonoids were found in all the extracts of the two plants [6]. The flavonoids present in these extracts could be the basis for the better activity demonstrated by these extracts because flavonoids have been considered to possess significant anti-inflammatory properties, both in vitro and in vivo [40,41]. Ethyl iso-allocholate found in all the extracts was reported for its anti-inflammatory activity [35]. Several authors, such as Lee et al. [42], proved the anti-inflammatory activity of *M. charantia*. Indeed, these authors showed that LPS-induced phosphorylation and nuclear translocation of NF-κB may be inhibited in the presence of *M. charantia*. Ayertey et al. [43] have shown, by using macrophages, that the anti-inflammatory effect of *M. lucida* may be due to inhibition of pro-inflammatory mediators, such as serotonin and histamine, inhibition of pro-inflammatory cytokines (IL-1b and TNF-a), inflammatory enzyme expressions (iNOS and COX-2) and their products (NO and PGE2). These authors [43] also established that *M. lucida* extracts attenuated systemic inflammation (with fever) induced by LPS in rats and upregulated the anti-inflammatory cytokine, IL-10. Another study [44,45] demonstrated that cucurbitane-type triterpenoids compounds isolated from *M. charantia* could inhibit the production of IL-6 in LPS-stimulated bone marrow-derived dendritic cells, and the authors attributed these anti-inflammatory effects to interactions of hydrogen bonds between the protein residues and hydroxyl groups and sugar rings of triterpenoids compounds.

There is an intrinsic relationship between diabetes, oxidative stress and inflammation [46]. Indeed, the antioxidant activity of the extracts of the two plants was evaluated in vitro by different methods. The extracts showed good antioxidant activity with ABTS and FRAP methods. Ayertey et al. [43] reported that *M. lucida* extracts showed a strong antioxidant property compared to the positive control. In our study, cyclic voltammetry was used to evaluate the reversible antioxidant effect of *M. charantia* and *M. lucida*. Electrochemical methods, such as voltammetry and amperometry, have been used to determine the total antioxidant capacity of the extracts [47,48,49]. The open circuit potential (OCP), which measures the equilibrium potential at the electrode surface, can be used to quantify a samples’ redox potential, which is a function of its composition [50]. It was registered in this study and the results obtained with *M. charantia* and *M. lucida* indicate that the overall redox potential of both plant extracts in methanol have no significant difference.

Cyclic voltammetry (CV) can yield much more information about multiple species in the electrolyte than OCP by qualitative or quantitative determination [48]. The antioxidant capacity of the extract is highlighted by its redox profile from CV. The anodic area of CVs can be correlated with the antioxidant capacity of the extracts [51]. Both anodic areas of cyclic voltammograms show the total content of antioxidant compounds, allowing discrimination between different extracts. We can conclude that the extracts undergo an irreversible oxidation in the same conditions for the anodic peak. The CV technique can also be helpful to determine the mechanism of free radical scavenging because of their potential use in traditional medicine.

## 4. Materials and Methods

### 4.1. Ethnopharmacological Study Area

The study was carried out in the form of surveys, which were conducted in southern Benin in the departments of Ouémé and Plateau. The study area belongs to the Guinean zone, which is located between 6°25′–7°30′ N and 2°33′–2°58′ E, where the rainfall regime is bimodal (April-June and September-November), with an average rainfall of 1200 mm per year. The average temperature varies from 25 °C to 29 °C and the air humidity from 69% to 97%. The vegetation formations of the area have been strongly affected by various agricultural activities and now form a mosaic of cultivated land and some relics of forest [52]. Specifically, the department of Ouémé is characterized by ferralitic, clayey–sandy, alluvial and colluvial soils, with essentially entropized vegetation made up of a few forest relics in places. There is also a grassy savannah, meadows, marshy raffia formations and some mangroves. The Plateau department is characterized by tropical ferruginous soils, bar land on the continental terminal and very deep and humus-rich clay soils. The climate is of the Sudano-Guinean type [53].

The populations of the study area are mainly composed of the Fon, Goun, Ouémè, Yoruba, Nago and Tori ethnic groups. They are mostly farmers whose main crops are maize, cassava, groundnut, cowpea and palm oil. They also engage in trade, fish farming and animal breeding.

### 4.2. Ethnopharmacology Survey

The ethnopharmacological survey was conducted among five ethnic groups (Goun, Ouémè, Yoruba, Tori and Sèto) of the selected villages. The choice of villages and ethnic groups was made according to a few considerations, such as: the accessibility of the area (village), the level of use of these plants in the broad sense, the socio-cultural particularities and the openness and adhesion of traditional healers and herbalists to participate in the survey. The respondents (traditional healers and herbalists) were sampled according to the method used by Assogbadjo et al. [54]. It consisted of addressing a question to 30 individuals from each ethnic group. The question was whether the individual knew both *M. charantia* and *M. lucida*. The sample size was determined by the binomial distribution formula described by Dagneli [55].

$$n=\frac{U_{\left(1-a\right)/2}^{2}\times p\left(1-p\right)}{d^{2}}$$

*n* is the sample size, p is the proportion of respondents who gave a positive answer (yes), *d* is the margin error of the evaluation and U1−/2 is the value of the random variable for probability 1−/2. For a probability of 0.975 (or = 0.05), U1−/2 ≈ 1.96. The ethnopharmacological surveys were conducted according to the methodology described by Legba et al. [56] and include semi-structured free surveys. The free semi-structured surveys were carried out in the form of open interviews with herbalists and traditional healers using a pre-established form. The free surveys were conducted both individually and in groups, while semi-structured surveys were conducted only in groups. In both types of survey, occasional discussions were also conducted for additional information. Various information on knowledge, the diseases treated, the recipes, the organs used, the dosages, the side effects and contraindications of the two plants under study (*M. charantia* and *M. lucida*) were collected and documented during the investigations in-field.

### 4.3. Biological Activity

#### 4.3.1. Chemicals

The extraction solvents, ammonium salt [2,2-azinobis (3-ethylbenzothiazoline-6-sulfonic acid)] (ABTS), phosphate bovine serum (PBS), bovine serum albumin (BSA), Butylhydoxytoluene (BHT) and ascorbic acid, were purchased from Sigma Aldrich (Steinheim, Allemagne). Diclofenac was obtained from Symed Pharm. Pvt., Ltd., Hyderabad, India. All the other chemicals and reagents used were of analytical reagent grade.

#### 4.3.2. Plant Material

The leaves of the plants used were locally grown. The *M. lucida* leaves samples were collected from Agata (06°30′28″ N, 002°38′44″ E), which is located in the department of Oueme, Benin, while those of *M. charantia* were collected from Dangbo (06°35′19″ N, 002°33′15″ E) located in the same department. The fresh samples collected were sent to the national herbarium. The samples were identified by the Researcher (Prof. YÉDOMONHAN Hounnankpon) in charge of the identification of plant species at the national herbarium of Benin. Voucher specimens No. AAC8100/HNB and No. AAC8101/HNB for *M. lucida* and *M. charantia*, respectively, were deposited at the Benin national herbarium, University of Abomey-Calavi, Cotonou, Benin. All the samples were collected in June 2022 in the morning at 7 a.m. They were air-dried (23 ± 2 °C) for two weeks before being powdered using a grinder Retsch-type SM 2000/1430/Upm/Smf, Haan, Germany.

#### 4.3.3. Preparation of Plants Extracts

The samples were prepared by extraction with different polar solvents (water, water–ethanol 30:70 (*v*/*v*) methanol, methanol/1% HCl, ethanol, acetone, ethyl acetate and dichloromethane) and non-polar solvents (chloroform and petroleum ether). For the polar solvents, 1 g of powder in 100 mL of solvent was subjected to ultrasonication (35 kHz) at room temperature for 2 h. The same operation was carried out with non-polar solvents under the reflux system. A total of 24 extracts were thus obtained, 12 per plant. In addition, the residues obtained after the ethyl acetate and petroleum ether extractions were extracted again using methanol and methanol/1% HCl, respectively. These extracts were coded Methanol-EA and Methanol/HCl-PE. Each mixture was filtered through Whatman N° 1 paper (125 mm ø, Cat No. 1001 125) and concentrated under reduced pressure using a rotary evaporator before being oven dried at 40 °C. The aqueous extract was lyophilized to dryness.

#### 4.3.4. Antioxidant Activity of Extracts by the ABTS Essay

The antioxidant activity was carried out according to the protocol described by Cudalbeanu et al. [57]. It is based on the discoloration of the stable radical cation ABTS+· [2,2′-azinobis-(3-ethylbenzothiazoline-6-sulfonic acid)] into ABTS. The ABTS radical cation stock solution was prepared by mixing an equal quantity (5 mL) of a 7.8 mM solution of ABTS and a 140 mM potassium persulfate solution. The mixture was kept in the dark for 12 h at room temperature. The solution was then diluted to obtain an absorbance between 1.1 ± 0.02 units at 734 nm. Fresh ABTS radical cation solutions were prepared for each run. A quantity of 100 µL of each extract at 500 µg/mL was mixed with 100 µL of the ABTS solution in a 96-well microplate. The same operation was realized for ascorbic acid (250 µg/mL) used as a reference. The absorbance was then measured at 734 nm after 30 min of incubation. All the assays were performed in triplicate.

#### 4.3.5. Ferric Reducing Antioxidant Power (FRAP) Essay

The reducing power was determined according to the method of Chio et al. [58]. Briefly, various concentrations of the extracts in 0.5 mL samples were mixed with 1 mL of a phosphate buffer (0.2 M, pH 6.6) and 1 mL of 1% potassium hexaferricyanide [K_3_Fe(CN)_6_], and the mixture was incubated at 50 °C for 30 min. Afterwards, 1 mL of 10% trichloroacetic acid was added to the mixture, which was then centrifuged at 3000× *g* for 10 min. Finally, 1 mL of the upper layer of the solution was mixed with 0.2 mL of 0.1% FeCl_3_, the mixture was left to rest away from light and the absorbance was measured at 700 nm. The same operation was realized with BHT (0–100 µg/mL) used as a reference. The antioxidant activity linked to reducing power was expressed as antioxidant power (AP) following the formula

$$\mathrm{AP}=\frac{\mathrm{Abs}\;\mathrm{Extract}-\mathrm{Abs}\;\mathrm{blank}}{\mathrm{Abs}\;\mathrm{Extract}}\times 100$$

#### 4.3.6. Evaluation of the Reversible Effects of the Antioxidant Activity of *M. charantia* and *M. lucida* Extracts by the Cyclic Voltammetry Technique

Electrochemical investigations using cyclic voltammetry (CV) were used to assess possible reversible effects of the antioxidant activity of *M. charantia* and *M. lucida* leaf extracts. The electrochemical system consisted of an electrochemical cell (20 mL) with three electrodes: a carbon glass electrode as a working electrode, Ag/AgCl_sat_ (E^0^ = 0.194 V/NHE) as a reference electrode and a Pt wire as a counter electrode. The measurements were carried out at room temperature with the Bio-logic SP-150 galvanostatic potentiostat (Claix, France). The applied potential was E = ±1 V vs. Ag/AgCl_sat_, varying the scan rate between 10 and 200 mV/s. The working electrode was polished with the BASi^®^ polishing kit (alumina and diamond sludge) followed by washing with methanol after each voltammetry experiment. Quercetin was also analysed as the main component of the extracts. Fresh solutions of *M. charantia* and *M. lucida* leaf extracts at 1 mg/mL and quercetin (10^−3^ M) were prepared in methanol. UV-Vis spectra were recorded before and after the cyclic voltammetry experiments using the Specord 210 Plus dual-beam spectrophotometer (Analytik Jena, Jena, Germany). The spectra were performed in the wavelength range of 200–700 nm using 1 cm quartz cells.

#### 4.3.7. Anti-Inflammatory Activity by Inhibiting Protein Denaturation

The anti-inflammatory effect of the extracts of the two plants was evaluated in vitro by looking for the thermal denaturation of albumin by an adaptation of the protocol described by Sangita et al. [59]. The reaction mixture (250 μL) consisted of 10 μL of albumin, 140 μL of phosphate buffer (PBS, pH 6.4) and 100 μL of extracts of 20 mg/mL concentrations. In a 96-well microplate, a series of 10 successive dilutions (1/2) of each extract was made from sample solutions at 20 mg/mL dissolved in methanol or water. The mixtures were incubated in the dark for 15 min and then heated at 70 °C for 5 min. After cooling, their absorbance was measured at 660 nm against a blank prepared under the same reaction conditions by replacing the extracts with the same volume of solvent. Diclofenac (0-100 µg/mL), an anti-inflammatory, was used as the standard. For each concentration, three (03) tests were carried out. The effect of the extracts on the thermal denaturation of albumin at 70 °C was expressed by the inhibition rate, calculated according to the formula below

$$\mathrm{Inhibitory}\;\mathrm{rate}=\frac{\mathrm{AbsC}-\mathrm{AbsE}\;\times 100}{\mathrm{AbsC}}$$

AbsC: variation of absorbance at 660 nm of control, AbsE: variation of absorbance at 660 nm of the extracts.

### 4.4. GC-MS Analysis

This analysis was carried out to search for and identify the volatile compounds contained in two types of extracts from each plant. These are the ethyl acetate and acetone extracts of the two plants (*M. charantia* and *M. lucida*). After dissolving the extracts in methanol (1 mg/mL), the extract solution was filtered using a 0.45-µm diameter PTFE filter. GC-MS analyses were performed on a Varian 4000 electron impact mass spectrometer using a Varian CP-8400 injector. The 30 m × 0.25 mm-Factor four column capillaries had a particle size of 0.25 µm (Varian). The injection volume was 100 µL with the injection temperature set at 250 °C. The flow rate of helium gas through the column was 1 mL/min, the ions were generated at an electron impact (EI) of 70 kV, the temperature of the ion source was set at 200 °C and the mass range was *m*/*z* 50–1000. The column temperature was kept isothermal at 70 °C for 2 min and then raised to 300 °C at a rate of 10 °C/min. Authentic assay standards were used to compare the retention time and retention index with the detected compounds. The compounds were identified against the baseline mass spectra (National Institute of Standard and Technology Mass Spectral v2.1).

### 4.5. Data Processing and Statistical Analysis

The data from the field surveys were coded and entered into an Excel 2007 database. These data were analysed with SPSS software (Statistical Package for the Social Sciences) version 16.0 to determine the descriptive statistics in terms of percentage and mean. The consensus factor (Fic) was used to assess the degree of homogeneity of information [23]. It is calculated by the formula

(1) $$\mathrm{Fic}=\frac{\mathrm{Nur}-\mathrm{Nt}}{\mathrm{Nur}-1}$$

with Nur being number of reported uses per disease category and Nt being total number of species used for treatment. The values range from 0 to 1, where 1 indicates the highest level of consent.

The experimental results were presented as the mean ± standard deviation (SD) of three parallel measurements. The graphs were presented using GraphPad Prism 7.00 software. A multivariate variance analysis followed by Duncan’s test was performed. Values of *p* < 0.05 were considered statistically significant.

## 5. Conclusions

Several actions falling within the framework of the valorisation of *M. charantia* and *M. lucida*, two plants used within the Beninese population, have been undertaken. An ethnopharmacological survey made it possible to identify endogenous knowledge related to the traditional use of these two plants in the treatment of infectious and metabolic diseases. The volatile molecules contained in the leaves of *M. charantia* and *M. lucida* have been researched. This analysis revealed the richness of the extracts of the two plants in volatile compounds with variable relative abundances. The antioxidant activity assessed by the FRAP method was more remarkable than that of the ABTS radical reduction. The biological potential through the anti-inflammatory activity of the leaf extracts of *M. charantia* and *M. lucida* has given good results.

## Figures and Tables

**Table 1:** Frequency of the diseases treated with *M. charantia* and *M. lucida*.

Diseases Group	Diseases/Symptoms	Frequency (%)		Fic
		*M. charantia*	*M. lucida*	
Digestive system diseases	Ulcer	9.48	3.64	0.77
	Stomach pain in children	-	16.05	
	Constipation	24.08	-	
	Diarrhea	12.40	9.48	
Microbial and parasitic disease	Measles	100	-	0.96
	Malaria	62.04	93.43	
	Genital infection	-	10.21	
Gynaecological diseases	Sticky menstruation	52.55	-	0.98
	Painful menstruation	-	10.21	
	Postpartum lactation problem	8.75	-	
	Primary infertility	-	20.43	
	Early menopause	-	14.59	
Blood-related diseases	Hypertension	16.05	8.02	0.99
	Diabetes	71.53	56.93	
	Anaemia	-	13.13	
Others	Jaundice in newborns	59.85	-	
	Dermatological problem (wounds)	98.54	2.91	
	Muscle pain	29.92	-	
	Inflammation	29.92	10.94	
	Immunity boost	45.25	24.08	
	Abdominal pain	-	74.45	

Fic: Consensus factor.

**Table 2:** Type of recipe and *M. lucida* organs used for the treatment of pathologies.

Diseases/Symptoms	Type of Recipe	Solvent Used	Parts of Plants Used	Preparation Mode	Dosage	Duration of the Treatment
Ulcer	with *Parkia biglobosa* stem bark	water	leaves, root	maceration	1 glass 1/d	14 days
Stomach pain in children	only	water	root	maceration	¼ of glass 3/d	3 days
Diarrhea	with *Citrus aurantiifolia* (fruit)	water	leaves	trituration	1 teaspoon 1/d	until satisfied
Malaria	only	water	leaves	decoction	1 glass 3/d	3 days
Genital infection	with potash and sesame leaf	water	root, leaves	maceration	1 glass 1/d	1 week
Painful menstruation	only	water	leaves, bark	decoction	1 glass 1/d	period of menstruation
Primary infertility	with potash and *Carica papaya* leave	water	root	maceration	1 glass 2/d	10 days
Early menopause	only	palmier wine	leaves	decoction	½ of glass 2/d	30 days
Hypertension	only	water	leaves	decoction	1 glass 1/d	3 days
Diabetes	only	water	leaves	decoction or maceration	½ of glass 3/d	until normal test
Anemia	with *Xylopia aethiopica*	ethanol	leaves	maceration	½ of glass 1/d	3 days
Dermatological affection (wounds)	with *Ocimum gratissimum*	water	leaves	decoction	½ of glass 1/d	7 days
Inflammation	with *Cymbopogon citratus*	water	leaves, bark, root	decoction	1 glass 1/d	3 days
Immunity boost	only	ethanol	leaves	maceration	½ of glass 2/d	3 days
Abdominal pain	only	water	leaves, bark	decoction	1 glass 1/d	until satisfied

d: days.

**Table 3:** Type of recipe and *M. charantia* organs used for the treatment of pathologies.

Diseases/Symptoms	Type of Recipe	Solvent Used	Parts of Plants Used	Preparation Mode	Dosage	Duration of the Treatment
Ulcer	with *Vitelaria paradoxa*	Coca-Cola	whole plant	maceration	½ of glass 1/d	7 days
Constipation	only	ethanol	whole plant	maceration	½ of glass 1/d	2 days
Diarrhea	with *Citrus aurantiifolia* (fruit)	water	leaves	trituration	1 teaspoon 1/d	3 days
Measles	only	water	whole plant	trituration	½ of glass 1/d and pass over the body	3 days
Malaria	with *Annona multiflora*	water	whole plant	decoction	1 glass 3/d	3 days
Sticky Menstruation	only	water	leaves	trituration	½ of glass 1/d	2 days before menstruation
Postpartum lactation problem	with unripe fruit of *Carica papaya*	water	whole plant	maceration	1 glass 2/d	2 days
Hypertension	only	water	whole plant	decoction	½ of glass 1/d	3 days
Diabetes	only	water	whole plant	decoction	½ of glass 3/d	until normal test
Dermatological affection (wounds)	only	water	leaves	trituration	½ of glass 1/d and pass over the body	3 days
Muscle pain	only	ethanol	whole plant	maceration	½ of glass 2/d	3 days
Inflammation	with *Combretum micrabtum*	water	whole plant	decoction	½ of glass 1/d	3 days
Immunity boost	only	ethanol	leaves	decoction	½ of glass 3/d	3 days

d: days.

**Table 4:** Antioxidant activity of *M. charantia* and *M. lucida* extracts by ABTS test.

Solvents/Standard	Inhibitory Rate (%)	
	*M. charantia*	*M. lucida*
Ethanol	31.26 ± 1.32	48.27 ± 1.54
Methanol	3.86 ± 0.11	43.66 ± 2.01
Ethyl Acetate	50.13 ± 1.20	29.85 ± 1.86
Acetone	50.52 ± 0.88	4.63 ± 1.41
Dichloromethane	22.48 ± 1.45	43.40 ± 0.45
Chloroform	4.70 ± 1.37	-
Petroleum ether	-	-
Water	20.63 ± 0.28	13.75 ± 0.17
Methanol/HCl	42.14 ± 3.11	38.10 ± 2.14
Ethanol/water	34.24 ± 1.17	45.35 ± 1.28
Methanol/HCl-PE	43.12 ± 2.38	37.15 ± 2.65
Methanol-EA	12.35 ± 1.78	5.01 ± 1.47
Ascorbic Acid	75.23 ± 2.17	

Each value is the mean of three replicates ± SD.

**Table 5:** The reducing power of Fe^3+^ ions by extracts of *M. charantia* and *M. lucida*.

Solvents/Standard	*M. charantia*		*M. lucida*	
	% Inh	IC_50_ (mg/mL)	% Inh	IC_50_ (mg/mL)
Ethanol	59.46 ± 1.83 ^a^	<0.078	58.80 ± 0.96 ^a^	0.15 ± 0.06
Ethanol–water	60.75 ± 1.07 ^a^	<0.078	58.23 ± 1.26 ^ab^	0.21 ± 0.02
Methanol	58.98 ± 1.30 ^a^	<0.078	60.24 ± 0.56 ^a^	< 0.078
Ethyl Acetate	59.97 ± 0.064 ^a^	<0.078	59.61 ± 0.60 ^a^	0.10 ± 0.01
Acetone	60.14 ± 1.87 ^a^	<0.078	59.23 ± 0.22 ^a^	<0.078
BHT	80.12 ± 1.02	22.35 ± 1.15 (µg/mL)	-	-

%Inh: Inhibition percentage. The values followed by the same letter (^a^, ^b^) in the same column show no statistically significant differences (*p* > 0.05). Each value is the mean of three replicates ± SD.

**Table 6:** Albumin denaturation inhibition rate and IC_50_ values of *M. charantia* and *M. lucida* extracts.

Solvents/Standard	*M. charantia*		*M. lucida*	
	% Inh	IC_50_ (mg/mL)	% Inh	IC_50_ (mg/mL)
Ethanol	99.13 ± 0.12 ^a^	1.16 ± 0.04	57.32 ± 1.35 ^a^	1.72 ± 0.23
Methanol	99.53 ± 0.08 ^a^	1.08 ± 0.11	97.67 ± 2.02 ^b^	1.08 ± 0.04
Ethyl Acetate	97.22 ± 1.02 ^a^	0.22 ± 0.01	93.62 ± 1.13 ^c^	0.47 ± 0.01
Acetone	97.02 ± 1.35 ^a^	0.55 ± 0.01	97.19 ± 0.45 ^b^	2.02 ± 0.01
Dichloromethane	98.95 ± 0.89 ^a^	0.10 ± 0.02	98.34 ± 0.12 ^b^	0.37 ± 0.14
Chloroform	97.67 ± 0.19 ^a^	0.38 ± 0.01	96.29 ± 0.89 ^b^	0.12 ± 0.02
Water	93.09 ± 1.17 ^b^	2.03 ± 0.95	98.13 ± 0.09 ^b^	0.11 ± 0.01
Diclofenac	98.30 ± 0.76 ^a^	13.33 ± 0.76 (µg/mL)	-	-

%Inh: Inhibition percentage. The values in the same column, which are followed by different letters (^a^, ^b^, ^c^) show statistically significant differences. Each value is the mean of three replicates ± standard deviation (SD).

**Table 7:** GC-MS analysis of volatiles compounds identified in *M. charantia* and *M. lucida* extracts.

N°	Retention Time (min)	Volatile Compounds	Relative Abundance (%)			
			*M. charantia*		*M. lucida*	
			EAC	Act	EAC	Act
1	8.8	1, 12-Dicarbadodecarboran-2-amine, N-(4-methoxyphenyl)	0.37	0.16	0.54	-
2	9.11	Inconnu	0.58	-	-	-
3	9.56	2-(Allyloxy)-1,5-ditert-butyl-3-chlorobenzene		15.98	14.93	8.6
4	10.51	Dibenzoic[c,H] diazecine, 6,13-bis (2,5-dimethylphenyl)-5,6,7,12,13,14-hexahydro-1,4,8,11-tetramethyl	-	-	0.4	0.42
5	10.73	Harzianic acid	-	-	-	0.3
6	10.81	4,4,7-Trimethyl-5-[(4H-1,2,4-triazol-3-ylsufanyl)acetyl]-4,5-dihydro-1H-[1,2]dithiolo [3,4-c]quinoline-1-thione	-	-	1.39	-
7	10.89	Inconnu	-	-	9.68	-
8	10.92	2-[(5-Chloro-8-hydroxy-3-methyl-1-oxoisochroman-7-carbonyl)amino]-3-phenyl propionate	-	2.42	-	-
9	12.92	1,3,5,7-Tetraethyl-1-butoxycyclotetrasiloxane	0.68	-	-	-
10	13.67	*Trans*-4,4′-Dimethoxy-β-methylchalcone	-	-	5.34	-
11	13.68	17-Hydroxy-3,20-dioxopregna-1,4,9(11)-trien-21-yl acetate	-	12.29	-	20.02
12	14.83	Harzialactone	-	0.56	-	-
13	14.84	Ent-3-Acetoxy-10-hydroxy-13-iodomethyl-16-oxo-8,13-epi-17,20-dinorgibberell-1-ene-7,19-dioic acid 19,10-lactone	-	-	0.2	-
14	14.89	Inconnu	-	-	-	0.32
15	16.51	Methanethione, (2,5-dimethylphenyl)-(2,4,6-trimethylpheny)-S-oxide	0.29	-	-	-
16	21.74	4-Methylcholesta-8,24-dien-3-ol	0.19			
17	25.96	17-(2-Butyl-1,3,2-dioxaborolan-4-yl) androstane-3, 11-diol	0.48	-	-	-
18	26.14	Inconnu	1.43	-	-	-
19	26.16	2,6-Dimethyl-4-(methoxymethyl)phenol	-	0.79	0.47	0.61
20	26.57	2-5Cyano-ethoxycarbonylmethyl)-6-methyl-4,6-bis(4-nitrophenyl)-1,6-dihydropyridin-3-3-carboxylic acid, ethyl ester	0.43			
21	27.1	5,6-Epoxy-7-bromocholestan-3-ol	-	-	0.29	-
22	27.26	3-β-5-epoxy-3-α-methoxy-a-homo-5-β-cholestane	0.41	-	-	-
23	27.5	*Cis*-13,14-Epoxydocosanoic acid	0.96	0.25	0.34	0.28
24	27.72	Androst-4-ene-3,20-dione, 11,16,22-triacetoxy-	0.24	-	-	-
25	29.49	Lycopene, 3,3′,4,4′-tetradehydro-1,1′,2,2′-tetrahydro-1-hydroxy-1′-methoxy-	0.33	-	-	-
26	29.54	2-Chloroethyl isobutyrate of terephthalate	-	-	-	0.34
27	29.55	6,9,12,15-Docosatetraenoic acid, methyl ester	-	-	1.14	-
28	29.67	3,5-Androstadien-17-one oxime	0.49	-	-	-
29	29.71	2-Bromo-4,6-dimethylbenzamide	-	-	-	0.51
30	29.56	17-Acetyl-16-hydroxy-10,17-dimethylgona-4,13-dien-3-one	-	0.1	-	0.07
31	30.18	Lycopene, 3,4-didehydro-1,2-dihydro-1-methoxy-,all-*trans*	0.12	-	-	-
32	30.32	Inconnu	-	10.53	0.38	0.40
33	32.96	Propanoic acid, 2-(3-acetoxy-4,4,14-trimethylandrost-8-en-17-yl)	7.03	-	2.23	-
34	33.05	Pregn-16-en-20-one, 11,18-bis (acetyloxy)-3,9-epoxy-3-methoxy	-	0.53	-	0.1
35	33.38	(2-Phenyl-1,3-dioxolan-4-yl)methyl (9E)-9-octadecenoate	0.23	0.37	-	-
36	33.4	Galoxolide	-	-	2.1	3.58
37	34.36	3-(3-Bromophenyl)-7-chloro-10-hydroxy-3,4-dihydro-1,9 (2H, 10H)-acridinedione	0.16	-	-	-
38	35.13	3,9,14,15-Diepoxypregn-16-en-20-one, 3,11,18-triacetoxy	11.26	-	-	-
39	35.17	3,9,14,15-Diepoxypregn-16-en-20-one, 3,11,18-triacetoxy	-	-	-	1.85
40	35.47	17-(1,5-Dimethylhexyl)-10,13-dimethyl-3-styryhexaderhydrocyclopenta[a]phenanthren-2-one	0.79	-	-	-
41	35.63	5-Stigmastane-3,6-dione	-	1.25	-	0.58
42	35.85	1-Heptatriacontanol	0.68	2.66	1.06	0.7
43	36.66	Aromadendrene	0.59	10.52	-	0.51
44	37.01	Inconnu	6.26	-	-	-
45	37.02	6β-Hydroxyfluoxymesterone		3.24	15.55	14.31
46	37.46	Inconnu	-	-	2.62	-
47	37.83	Ethyl iso-allocholate	14.33	2.42	0.68	3.63
48	37.99	6-Hydroxyfluoxymesterone	-	3.69	-	-
49	38.00	2,6-Bis (1,1-dimethylethyl)-4-(1-oxopropyl)phenol	27.03	2.55	3.18	3.17
50	38.63	Inconnu	1.15	-	-	-
51	38.77	Dermadine	2.54	12.66	2.86	-
52	38.79	17-Ethylenedioxy-5,19-cycloandrost-6-en-3-one	-	-	-	2.86
53	39.26	(22S)-21-Acetoxy-6,11-dihydroxy-16,17-propylmethylenedioxypregna-1,4-diene-3,20-dione	2.06	1.64	2.26	1.68
54	40.44	Inconnu	-	1.16	-	4.03
55	41.16	3,12,25-Tris(acetyloxy)cholestan-7-yl acetate	6.05	-	4.49	4.78
56	41.5	N,N’-Bis(Carbobenzyloxy)-lysine methyl (ester)	1.13	0.87	0.84	22.84
57	42.67	Harzianolide		12.39		
58	42.69	Benzothiophene-2-carboxylic acid, 4,5,6,7-tetrahydro-7-hydroximino-3-[2-(4-morpholyl)-1-oxoethylamino]-, ethyl ester	10.54	-	25.33	-
**Total**			98.83%	99.03%	98.30%	96.49%

EAC—ethyl acetate extract, Act—acetone extract.

## Data Availability

Not applicable.
